# Effects of m-Aramid/p-Aramid Blend Ratio on Tensile Strength due to UV Degradation for Firefighter Clothing Fabrics and Development of Predictive Equation for Tensile Strength

**DOI:** 10.3390/polym14163241

**Published:** 2022-08-09

**Authors:** Kaoru Wakatsuki, Minami Matsubara, Norimichi Watanabe, Limin Bao, Hideaki Morikawa

**Affiliations:** 1 Institute of Fiber Engineering, Shinshu University, Ueda 386-8567, Nagano, Japan; 2 Faculty of Textile Science and Technology, Shinshu University, Ueda 386-8567, Nagano, Japan

**Keywords:** ultra violet, tensile strength, high performance fiber, aramid, degradation, ageing

## Abstract

This study focused on the m-Aramid/p-Aramid blend ratio of the fabrics, clarified the quantitative relationship between UV exposure and strength retention, and developed a mathematical model to calculate tensile strength from an arbitrary amount of UV exposure energy. The results of tensile strength tests before and after UV exposure showed that the decrease in tensile strength due to UV degradation depended on the combination of p-Aramid and m-Aramid blend percentages. Tensile strength for all blend ratios decreased exponentially with UV exposure energy and was within the range of results for fabrics with p-Aramid 100% and m-Aramid 100%. The retention fraction of tensile strength, which represents the tensile strength after UV exposure relative to the initial tensile strength, decreased exponentially with increasing the fraction of UV exposure energy for all fabrics used in this study. Fitting the retention fraction of tensile strength to the fraction of UV exposure energy, two groups of fabrics were classified based on m-Aramid blends of 40% or more and 60% or less. This model can predict the tensile strength of firefighter clothing fabrics that retain high mechanical strength when exposed to UV light and design the strength of firefighter clothing with consideration of degradation over time.

## 1. Introduction

Firefighter clothing generally consists of three layers of fabric: an outer layer, a moisture barrier, and a thermal liner. The outer layer requires mechanical strength and flame/heat resistance and is made of aramid fibers, aramid fibers (p-Aramid and m-Aramid), and PBO (poly-(p-phenylene-2,6-benzobisoxazole)) or PBI (poly-2,2′-(m-phenylene)-5,5′-bisbenzimidazole) blended fibers. 

Aramid fibers are aromatic polyamide fibers defined as “those in which 85% or more of the amide groups (-NHCO-) are directly bonded to two or more aromatic rings, and 60% or more of the amide groups can be replaced by aromatic imide groups” and are classified into m-Aramid and p-Aramid, according to the position of the amide groups bonded to the aromatic ring [1]. Aramid fibers have a linear molecular structure containing aromatic rings, which gives them rigidity and thermal resistance. On the other hand, aramid fibers have an amide bond in their chemical structure, making them susceptible to mechanical strength degradation under ultraviolet light [2,3,4]. 

Performance requirements for firefighter clothing are currently ISO 11999-3 (firefighter clothing for building fires) [5], ISO 15538 (firefighter clothing with reflective outer layer) [6], and ISO 15384 (firefighter clothing for wildland fires) [7]. The mechanical strength performance of the outer layer of firefighter clothing is evaluated by tensile and tear strength. ISO 11999-3 specifies that an outer layer of firefighter clothing should have a tensile strength of 450 N or more at performance level b1 and 800 N or more at performance level b2 and have a tear strength of 25 N or more at performance level b1 and 40 N or more at performance level b2. These evaluation tests and performance requirements are for firefighter clothing fabrics before shipment and after exposure to heat but not after aging, due to UV exposure. The cautionary note for maintenance and management regarding UV exposure of firefighter clothing states: “Firefighter clothing shall be stored away from direct sunlight and UV exposure. To prevent the fabric of firefighter clothing from deteriorating due to UV exposure, keep it out of direct sunlight during drying.” Considering the conditions of use in actual firefighting activities and training and drying after washing, firefighter clothing is exposed to UV exposure caused by sunlight and flames. Therefore, although firefighter clothing has the mechanical strength of the value specified at the time of product shipment, firefighter clothing exposed to UV exposure with use does not necessarily meet the standard value for tensile strength. Many firefighters have experienced the ripping and tearing of firefighters clothing during firefighting activities.

According to a study on ultraviolet degradation of firefighter clothing by Davis et al. [8], the decrease in tensile strength resulting from exposure to ultraviolet light indicates that the outer layer of firefighter clothing does not have the required tensile strength. Despite such reports, there are very few examples of studies that contribute to a specific replacement time based on the tensile strength loss of firefighter clothing exposed to UV light. Ripping and tearing the outer layer of firefighters’ clothing during firefighting activities and training seriously compromises the safety of firefighters regarding their bodies and lives. However, the criteria for replacing firefighter clothing based on the tensile strength degradation caused by ultraviolet rays have not yet been clarified.

Said et al. [9], in their study on UV degradation of high-strength fibers, showed that the tensile strength of commercial yarns made of para-aramid and PBO high-performance fibers decreases with UV irradiation. Horikawa et al. [10] investigated the effect of UV irradiation on the tensile strength of PBO fibers using single fiber tensile tests, SEM, and AFM observations. They showed that surface roughness increased, and tensile strength decreased with increasing UV exposure. The factors affecting the tensile strength of PBO fibers exposed to UV were the UV exposure dose and the surface roughness due to autoxidation reaction in the non-crystalline part. 

Davis et al. [8] showed that UV irradiation causes deformation of the fiber surface and decreases the tensile strength of PPTA (Poly(p-phenylene terephthalamide)) fibers. The infrared spectroscopic analysis confirmed that UV irradiation caused the cleavage of amide bonds and the formation of oxidized species, suggesting that PPTA fibers undergo photo-oxidation. El Aidani et al. [11] investigated the changes in structural and mechanical properties of the moisture barrier layer of firefighter clothing made of m-Aramid fiber when exposed to ultraviolet light. The IR spectrum showed a new absorption band at 1725 cm^−1^ after UV irradiation due to carbonyl groups, suggesting a UV degradation mechanism for m-Aramid. Mechanical properties showed a decrease in tensile and tear strength after UV irradiation, and SEM observations showed that after UV irradiation, longitudinal cracking leads to fiber breakdown, exfoliation that generates micropores, and the formation of transverse cracks. Kamocka-Bronisz [12] conducted a tensile strength analysis of the outermost layer of firefighter clothing made of aramid fiber after accelerated aging and abrasion and reported a decrease in tensile strength after UV irradiation.

Yamaguchi [13] quantitatively estimated the strength of aramid fibers after UV irradiation by assuming that the number of defects in aramid fibers increases due to UV irradiation, based on Weibull’s weakest link theory regarding the degradation phenomenon of aramid fibers due to UV irradiation. Arrieta et al. [4] showed that the amount of carboxylic acid produced by the hydrolysis reaction could be estimated using the relative intensity corresponding to the aromatic group of the carboxylic acid for the degradation progression to hydrolysis of fabrics blended with p-Aramid and PBI fibers. The kinetic model was used to estimate the amount of carboxylic acid formed by hydrolysis. The kinetic model can formulate the degradation of hydrolysis. Rezazadeh et al. [14] showed that the tensile strength of a thermally degraded aramid fiber of firefighter clothing fabric could be estimated from its infrared reflectance using near-infrared spectroscopy. Ormond et al. [15] investigated the possibility of nondestructive testing of firefighter clothing outer layers by FTIR. Infrared absorption spectra of aramid fiber fabrics exposed to ultraviolet, washing, and radiant heat irradiation were measured to confirm the correlation between peak intensity and tear strength. Although these previous studies have modeled the degradation of individual fibers against UV exposure, no report universally handles the change in tensile strength with UV irradiation energy on the outer layer of firefighter clothing with varying blends of m-Aramid and p-Aramid.

Aramid fibers generally have excellent thermal, flame, and mechanical strength. Among them, m-Aramid has the advantages of high flame resistance and thermal resistance, while p-Aramid has the benefits of high mechanical strength and slight thermal shrinkage. However, both fibers are known to degrade under ultraviolet light. p-Aramid exhibits more significant strength degradation than m-Aramid. Therefore, optimizing m-Aramid and p-Aramid, according to the desired performance, determines the properties of the outer layer of firefighter clothing. Thus, the relationship between the tensile strength of the outer layer, due to UV degradation and the blend ratio of the fibers, should be clearly defined. 

This study experimentally evaluates and characterizes the effect of UV exposure on the tensile strength of the outer layer of firefighter clothing fabrics by using fabrics with different m-Aramid and p-Aramid blends. Next, this study models the tensile strength of outer layer firefighter clothing after UV exposure. It establishes a method for quantitatively estimating tensile strength for a given amount of UV exposure energy. This model enables us to easily predict the UV properties of outer layer fabrics of firefighter clothing that retain high mechanical strength by calculating and designing the strength of firefighter clothing with consideration of degradation over time.

## 2. Experiment

### 2.1. Test Specimen

Table 1 shows the type and specifications of the outer layer fabric of firefighter clothing used in this study. The two-ply spun yarns used for the fabrics were 100% m-Aramid, 100% p-Aramid, and a blend of m-Aramid and p-Aramid. Samples A, G, H, and I have a water-repellent finish on the fabric’s surface. All fabrics’ structure is plain weave with ripstop. Ripstop is a yarn woven in a grid pattern at regular intervals to prevent the fabric from ripping.

**Figure 1:**
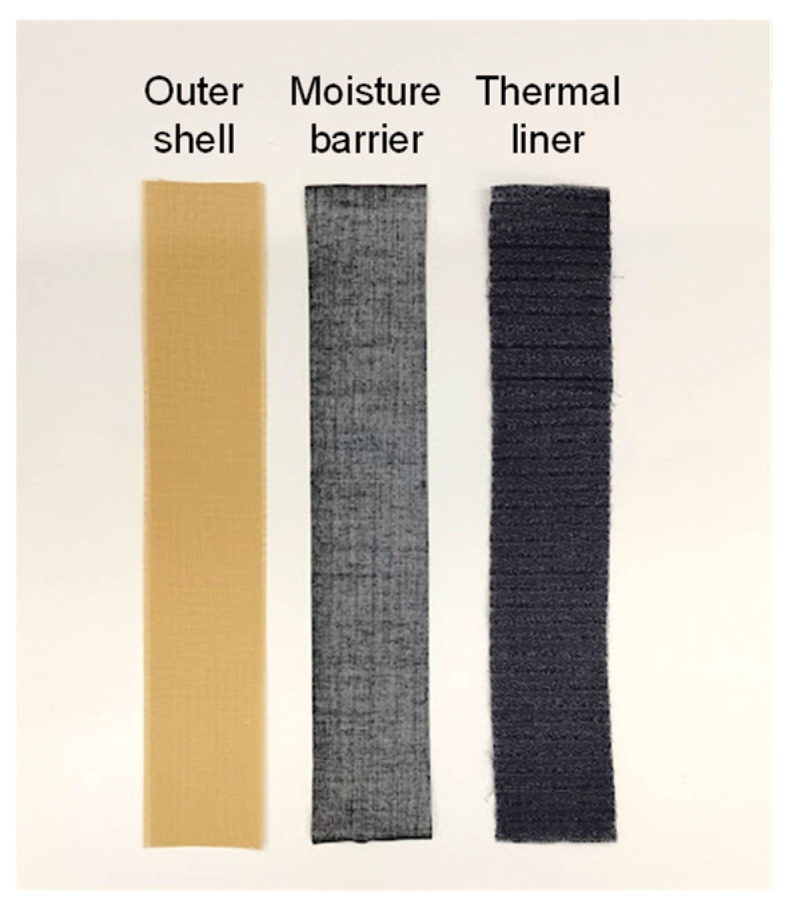
Example of firefighters’ protective clothing fabrics (Sample A).

Figure 1 shows an example of a test specimen (Sample A) used for UV exposure. The fabric specimens for UV exposure were layered in the order of outer layer, moisture barrier layer, and thermal liner to reproduce the actual structure of firefighter clothing. Tensile tests were performed on the outer layer specimens. The fabric specimens’ dimensions for UV exposure were based on ISO 13934-1 [16], with the warp direction of the outer layer, moisture barrier, and thermal liner being 300 mm long × 55 mm wide. For the outer layer, the warp direction was 300 mm long × 50 mm wide, with approximately the same number of warp threads taken from both ends to minimize irregularities’ impact on the tensile strength.

### 2.2. UV Exposure Procedure

UV exposure was conducted using a xenon-arc weathering meter (SX 75, Suga Test Instrument) with a light source whose wavelength of 300–400 nm closely approximates the spectral irradiance distribution of the ultraviolet and visible portions of sunlight. The radiation intensity of the xenon arc lamp was 180 W/m^2^. The temperature of the black panel thermometer in the chamber of the weather meter was 63 °C, and the relative humidity was 50% RH. Because this UV exposure focused on degradation caused simply by UV exposure, distilled water was not sprayed to reproduce actual weather conditions. 

**Figure 2:**
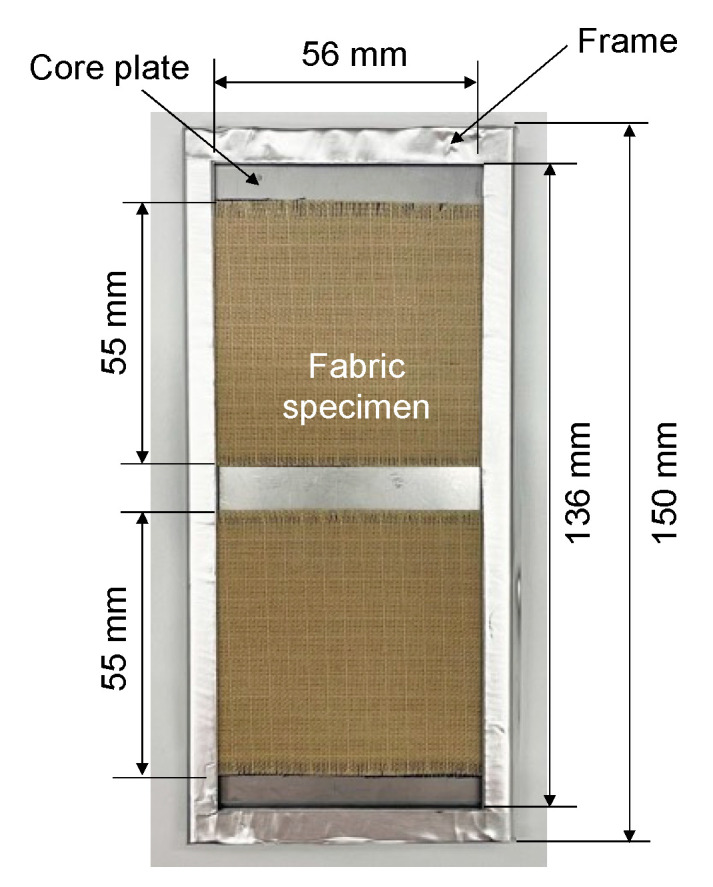
Test specimen placement in the test frame.

**Figure 3:**
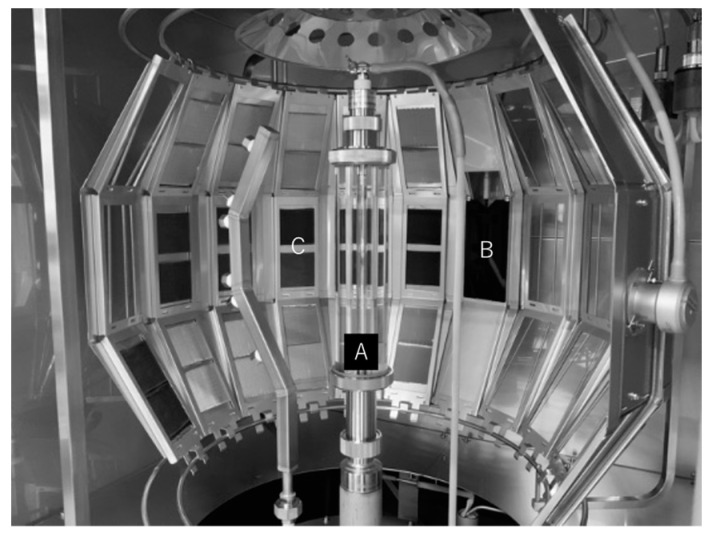
Example of sample holders’ placement in the xenon-arc weathering meter. The letters “A”, “B”, and “C” represent the xenon arc lamp, the black panel thermometer, and the test frame with two test specimens, respectively.

Figure 2 shows the test specimen mounted in the test frame with a core plate. The test specimens in the test frame with a core plate were assembled in the same order as the structure of firefighter clothing, from the outer layer, the moisture barrier layer, and the thermal liner. The outer layer was the first layer of UV radiation. The test frame with the specimens attached was periodically switched between the upper, middle, and lower positions in a specimen holder to minimize unevenness in the UV exposure conditions. The UV exposure area was 55 mm in the weft direction and 28 mm up and down from the center of the fabric in the warp direction (56 mm in total). Figure 3 shows an example of specimen holders’ placement in the xenon-arc weathering meter. The test frame was mounted in a specimen holder and installed in the weathering testing chamber. The letters “A”, “B”, and “C” in Figure 3 represent the xenon arc lamp, the black panel thermometer, and the test frame with two test specimens, respectively.

### 2.3. UV Exposure Conditions

UV exposure conditions were based on ISO 4892-2 [17] and JIS D 0205 [18]. The UV wavelength range (300–400 nm) accounts for 6.8% [17], considering only the UV radiation is mainly responsible for material degradation. The UV exposure energy in this wavelength range is 306 MJ/m^2^, compared to the annual average radiation exposure of 4500 MJ/m^2^ of sunlight in Japan.

The time of exposure of firefighter clothing to ultraviolet rays per year was determined from interviews with firefighters as follows. First, the period of exposure of firefighter clothing to ultraviolet light was set to 1/3 of a year, since firefighters work three 24 h shifts. Second, firefighters who used firefighter clothing had two hours per day for firefighting activities or training. Third, firefighter clothing was assumed to be washed and dried outdoors. The drying time was supposed to be 2 h per day. In other words, the total daily exposure of firefighter clothing to sunlight was assumed to be 4 h. Since Japan’s average daily direct solar radiation is 12.08 h per year [19], firefighter clothing is exposed to 1/3 of the daily direct solar radiation hours. Based on these assumptions, the exposure time of firefighters’ clothing to ultraviolet radiation was assumed to be 1/9 of a year. The UV energy at the wavelengths to which firefighter clothing is exposed in a year is supposed to be 34 MJ/m^2^. Table 2 shows the UV exposure conditions. The exposure time of the xenon arc lamps was set, based on Equation (1), to correspond to the UV exposure shown in the table.

(1) $$Exposure\;time\;\left[hours/year\right]=\frac{UV\;exposure\;energy\;per\;year\;\left[\frac{J}{m^{2}year}\right]}{Radiation\;intensity\;of\;xenon\;arc\;lamps\;\left[\frac{W}{m^{2}}\right]}\times \frac{1\;\left[hour\;\right]}{3600\;\left[sec\;\right]}\;=\frac{34\times 10^{6}}{180}\times \frac{1}{3600}\;=52.4\;\mathrm{hours}/\mathrm{year}$$

### 2.4. Tensile Strength Test

Tensile tests were conducted on the outer layer using a tensile test instrument (RTC-1250A, A&D) with a 5 kN load cell (UR-5kN-D, A&D) by ISO 13934-1 [16]. Parallel clamping jaws (AJ-JFM-5kN, A&D) were used to hold the specimens. The jaws were clamped with a 20 ± 2 N-m to prevent breakage within 1 cm of the jaw edge and specimen slippage from the jaws. The test conditions were a tensile speed of 100 mm/min, a distance of 200 mm between jaws, and a sampling rate of 0.01 s.

Tensile tests were conducted three times for each UV exposure condition, and the average value of the maximum tensile strength (N) was defined as the tensile strength of the specimens. The severity of degradation was defined as the retention fraction of tensile strength, which is the fraction of the tensile strength of the exposed specimen to the one of the unexposed specimens, as shown in Equation (2).

(2) $$Retention\;fraction\;of\;tensile\;strength\;\left[-\right]=\frac{Tensile\;strength\;of\;the\;exposed\;specimen\;\left[N\right]}{Tensile\;strength\;of\;the\;unexposed\;specimen\left[N\right]}$$

## 3. Results

Table 3 summarizes the tensile strength and retention fraction of tensile strength (*I/I_0_*) of the outer layer fabric of firefighter clothing blended with m-Aramid and p-Aramid. Based on these data, the relationships between tensile strength (*I*) to UV exposure energy (*Q*), the retention fraction of tensile strength (*I/I_0_*) to the fraction of UV exposure energy (*Q/Q_0_*) were analyzed. The fraction of UV exposure energy (*Q/Q_0_*) is calculated by dividing the UV exposure energy (*Q*) by the UV exposure energy per year (*Q_0_* = 34 MJ/m^2^), which corresponds to the number of years of exposure.

### 3.1. Effect of Water-Repellent Treatment on Tensile Strength and Retention Fraction of Tensile Strength

**Figure 4:**
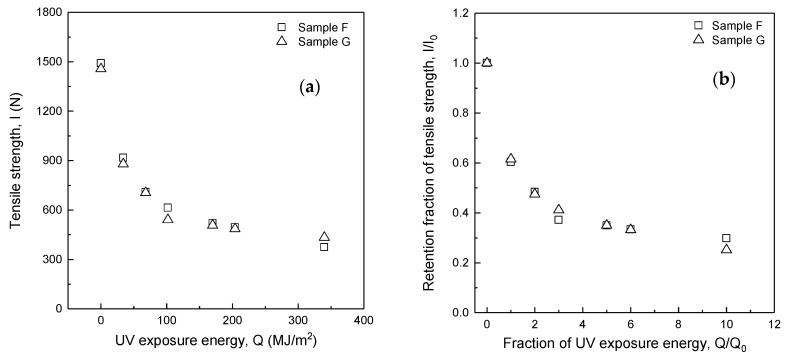
Tensile strength change (**a**) and retention fraction of tensile strength change (**b**) due to UV exposure with and without water repellency (m-Aramid 90%/ p-Aramid 10%).

Figure 4a,b show the relationship of the tensile strength (*I*) to UV exposure energy (*Q*) and the retention fraction of tensile strength (*I/I_0_*) to the fraction of UV exposure energy (*Q/Q_0_*) for Samples F and G, respectively. As shown in Table 1, Samples F and G have the same blend ratio (m-Aramid 90%/p-Aramid 10%), fabric mass, and fabric structure, but the difference is the presence of water-repellent treatment. As a result, the tensile strengths of Samples F and G have decreased exponentially with increasing UV exposure energy (*Q*), and the respective plots are mostly overlapped.

Table 4 shows the difference in tensile strength and the percentage difference between Sample G without water repellent treatment and Sample F with water-repellent treatment. The tensile strength and percentage differences are −72.5 N (−13.4%) and 58.7 N (13.5%) at UV exposure energies *Q* = 102 MJ/m^2^ and 340 MJ/m^2^, assuming 3 and 10 years of use of the firefighter clothing, respectively. However, the difference and percentage difference for the other exposure energies averaged −19.8 N (−2.4%). On the other hand, the differences in the retention fraction of tensile strength (*I/I_0_*) shown in Table 4 were 0.04 and −0.05 for a fraction of UV exposure energy *Q/Q*_0_ = 3 and 10, respectively, but were only 0.004 on average for the other fraction of UV energy exposed to the test specimen to UV energy per year.

The specimen fabrication and tensile test methods were correct because the percentage difference in initial strength between the specimens with and without water repellent treatment was −2.4%. In addition, the specimens were all taken from the same fabric, and the standard deviation of tensile strength for each UV exposure energy was slight. However, the possible reason for the results at *Q* = 102 MJ/m^2^ and 340 MJ/m^2^ could be the effect of specimen holder rotation, which regularly exchanges the locations of the holder at the top, middle, and bottom. Therefore, the water repellent treatment does not affect tensile strength for fabrics with the same blend ratio, fabric mass, and structure.

### 3.2. Effect of Fabric Mass on Tensile Strength and Retention Fraction of Tensile Strength

**Figure 5:**
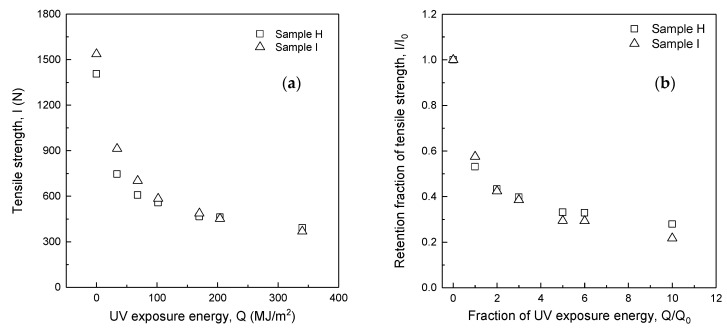
Tensile strength change (**a**) and retention fraction of tensile strength change (**b**) due to UV exposure with the same fiber blending ratio (m-Aramid 100%).

Figure 5a,b show the relationship of the tensile strength (*I*) to UV exposure energy (*Q*) and the retention fraction of tensile strength (*I/I_0_*) to the fraction of UV exposure energy (*Q/Q_0_*) for Samples H and I, respectively. As shown in Table 1, Samples H and I have the same blend ratio (m-Aramid 100%) but different fabric mass and number of ripstop. As a result, the tensile strength of Samples H and I have decreased exponentially with increasing UV exposure energy (*Q*). Table 5 shows the tensile strength and percentage difference between Samples H and I. At UV exposure energies from 0 MJ/m^2^ to 68 MJ/m^2^, the average tensile strength difference and percentage difference of Sample I relative to Sample H was 131.5 N (13.5%) higher. This result indicates that fabric mass difference strongly affects tensile strength in this UV exposure energy range. At UV exposure energies from 102 MJ/m^2^ to 170 MJ/m^2^, the average difference and percentage difference in tensile strength of Sample I relative to Sample H was 24.3 N (4.6%), while at 340 MJ/m^2^, it was −23.1 N (−6.3%). Since ripstop is applied to the fabric structure to prevent the fabric from tearing, the tensile strength difference is attributed to the fabric mass. As a result, the effect of fabric mass on tensile strength was significant for UV exposure energies from 0 MJ/m^2^ to 68 MJ/m^2^. Still, it became decreased with increasing UV exposure energy when the UV exposure energy exceeded 102 MJ/m^2^.

Table 5 and Figure 5b show the relationship between the fraction of UV exposure energy (*Q/Q_0_*) and the retention fraction of tensile strength (*I/I_0_*). The retention fraction of tensile strength (*I/I*_0_) for Samples H and I decreased exponentially with the increasing fraction of UV exposure energy (*Q/Q*_0_). The difference in the retention fraction of tensile strength for the Sample H with the lower fabric mass was 0.05 higher than Sample I when the UV exposure energy ratio was *Q/Q*_0_ = 1, i.e., when the firefighter clothing had been used for one year. However, the difference became −0.01 at *Q/Q*_0_ = 2 and 3, −0.04 at *Q/Q*_0_ = 5 and 6, and −0.06 at *Q/Q*_0_ = 10, respectively.

### 3.3. Effect of Blending Ratio on Tensile Strength and Retention Fraction of Tensile Strength

**Figure 6:**
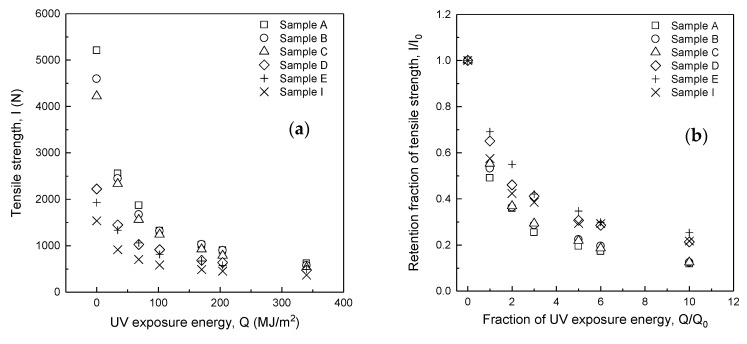
Tensile strength change (**a**) and retention fraction of tensile strength change (**b**) due to the change of p-Aramid/m-Aramid blending ratio.

Table 3 and Figure 6a show the relationship between tensile strength (*I*) and UV exposure energy (*Q*) for Samples A, B, C, D, E, and I. In common for all fabrics, tensile strength decreased exponentially with increasing UV exposure energy (*Q*) up to *Q* = 102 MJ/m^2^ and then moderately reduced. For example, comparing Sample A with p-Aramid 100% and Sample I with m-Aramid 100%, the difference in tensile strength before UV exposure was 3672.5 N, which was 3.4 times more significant, but the difference decreased as the UV exposure energy increased. For example, the difference at UV exposure energy *Q* = 340 MJ/m^2^, which assumes ten years of use of the firefighter clothing, decreased to 250.5 N, which was 1.7 times greater. This result indicates that the role of p-Aramid in increasing tensile strength gradually decreases as the UV exposure energy *Q* increases. 

Figure 6b shows the relationship between the fraction of UV exposure energy (*Q/Q*_0_) and the retention fraction of tensile strength (*I/I*_0_). The retention fraction of tensile strength (*I/I*_0_) decreases significantly in the early stages of UV exposure. For example, at a fraction of UV exposure energy (*Q/Q*_0_) of 3, i.e., *Q* = 102 MJ/m^2^, assuming three years of use of the firefighter clothing, the retention fraction of tensile strength (*I/I*_0_) was below 0.5 for all fabrics for any blend ratio of aramid. This result indicates that the tensile strength has decreased by more than 50% relative to the initial tensile strength. Fabrics blended with a high percentage of p-Aramid showed a lower retention fraction of tensile strength after UV exposure than fabrics blended with a high rate of m-Aramid. The retention fraction of tensile strength (*I/I*_0_) of Sample A and I with p-Aramid 100% and m-Aramid 100% were 0.25 and 0.42, respectively, at a UV exposure energy ratio (*Q/Q*_0_) of 3, and 0.12 and 0.22, respectively, at a UV exposure energy ratio (*Q/Q*_0_) of 10. These results indicate that the tensile strength of p-Aramid decreases faster under UV light than m-Aramid. The retention fraction for all fabrics is between Sample A and Sample I. Therefore, the decrease in tensile strength for fabrics blended with p-Aramid and m-Aramid can be predicted by combining the characteristics of the strength decrease (*I/I*_0_) relative to the fraction of UV exposure energy (*Q/Q*_0_) for p-Aramid and m-Aramid, respectively. 

The cause of mechanical characteristics change after UV exposure on aramid fibers has been analyzed by chemical structure, crystallinity, molecular weight, and surface characterization of the fiber structure. Yamaguchi [13] has shown that the fracture of para-aramid fibers originates from defects in the amorphous part of the fiber, where the bonding strength is weak, and has derived a quantitative relationship between the number of defects and tensile strength. This relationship was qualitatively the same as the relationship between UV exposure energy and tensile strength of the fabric from this research. However, Yamaguchi [13] has not investigated the quantitative relationship between tensile strength and the number of defects in m-Aramid fibers induced by UV exposure. Different approaches have been used to analyze the fracture mechanism of aramid fibers based on chemical structure, crystallinity, molecular weight, and surface characterization of the fiber structure. Still, the quantitative relationship for tensile strength can be consequently attributed to the number of defects resulting from chemical and physical changes within the fiber.

The results in this research are from a tensile test of the fabric in the warp direction. The tensile strength characteristics of fabrics are quantitatively similar to those of warp yarns. Therefore, the tensile strength characteristics of the fiber, the spun yarn, and the fabric are qualitatively the same. Thus, it is necessary to investigate the quantitative relationship between the number of defects in m-Aramid fibers and the reduction in strength caused by UV exposure and to relate the number of defects to the strength of each fiber when blended with p-Aramid and m-Aramid fibers.

### 3.4. Estimation of the Retention Fraction of Tensile Strength after UV Exposure

**Figure 7:**
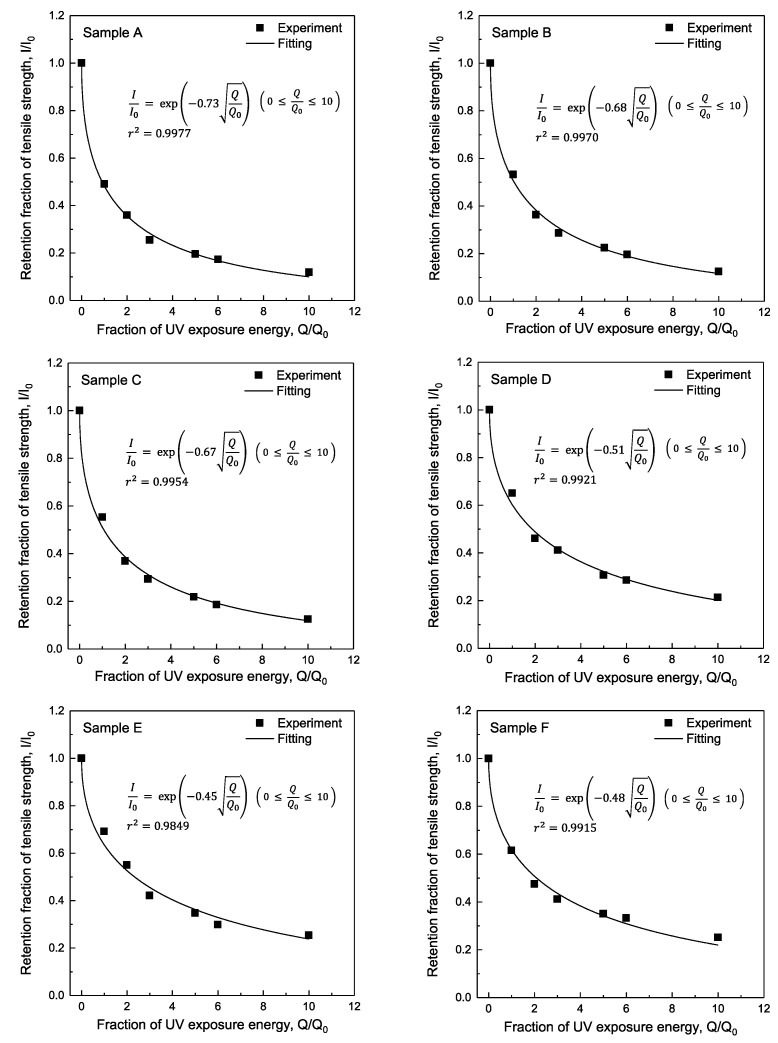

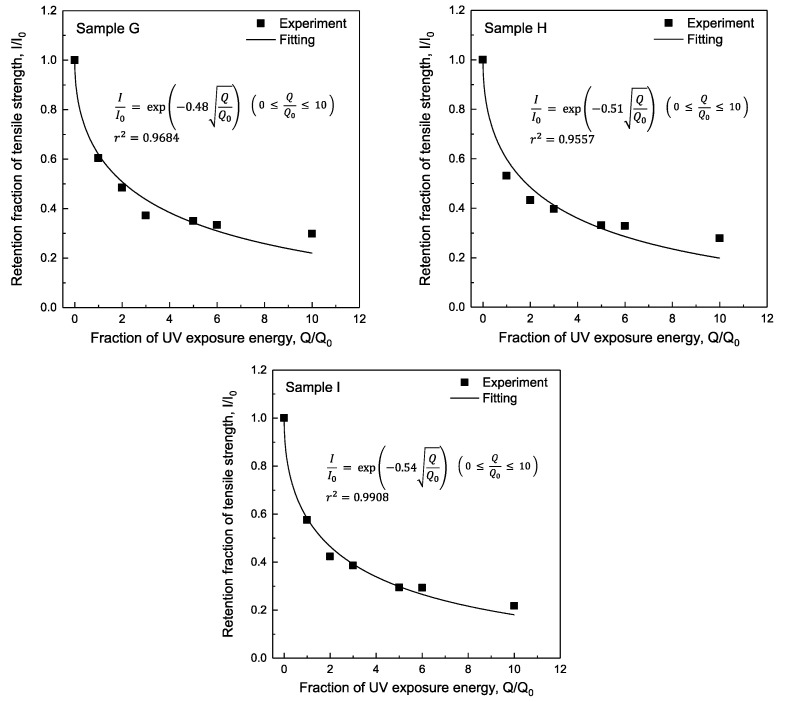
Relationship between a fraction of UV exposure dosage and retention fraction of tensile strength of Samples A to I. Symbols represent experimental data, and a solid line represents the fitting data by Equation (3).

Figure 7 shows the relationship between the fraction of UV exposure energy (*Q/Q*_0_) and the retention fraction of tensile strength (*I/I*_0_) for the p-Aramid and m-Aramid blended fabrics used in this study and the curve-fitting results. The retention fraction of tensile strength decreases exponentially with an increasing fraction of UV exposure and can be summarized as $\sqrt{Q/Q_{0}}$. Equation (3) shows the relationship between the fraction of UV exposure energy (*Q/Q*_0_) and the retention fraction of tensile strength (*I/I*_0_) obtained by curve fitting.

The degradation coefficient *α_f_* is higher for fabrics whose tensile strength decreases rapidly due to UV exposure.

(3) $$\;\frac{I}{I_{0}}=\exp\left(-\alpha_{f}\sqrt{\frac{Q}{Q_{0}}}\right)\;\;\;\;\;\;\;\;\left(\;0\;\leq \;\frac{Q}{Q_{0}}\;\leq \;10\;\right)$$

**Figure 8:**
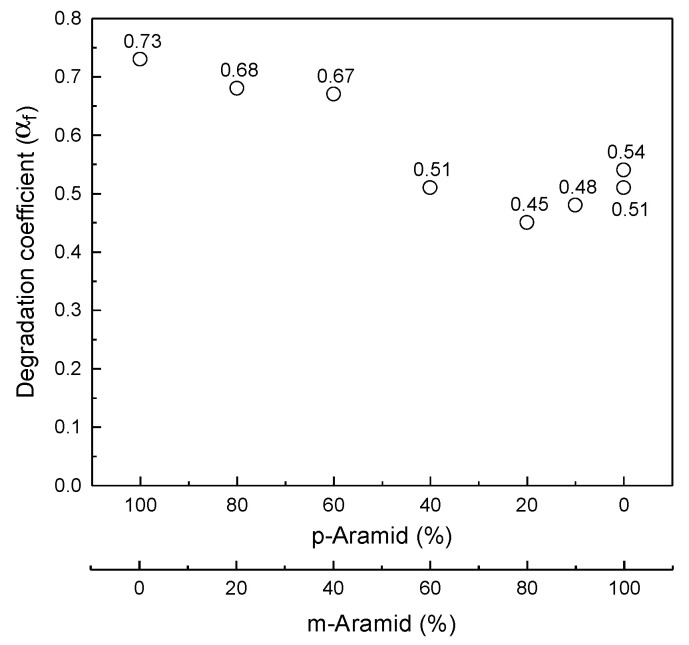
Relationship between degradation coefficient (α_f_) of the fitting Equation (3) and blend of p-Aramid/m-Aramid.

Figure 8 shows the distribution of the degradation coefficient *α_f_* for the p-Aramid and m-Aramid blends in the samples used in this study. The degradation coefficients *α_f_* for Sample A with p-Aramid 100% and Sample H with m-Aramid 100% are 0.73 and 0.51, respectively, indicating that the degradation coefficients for all fabrics are within this range. The degradation coefficient was estimated to be a linear combination of the degradation coefficient of p-aramid 100% and m-aramid 100% and the respective blend ratio, but the analysis results were divided mainly into two groups: m-aramid blend ratio of 40% or less and 60% or more. The average value of the degradation coefficient *α_f_* was 0.7 for fabrics with m-aramid blends of 40% or less, i.e., aramid blends that emphasize strength. The average value of *α_f_* was 0.5 for fabrics with m-aramid blends of 60% or more, i.e., aramid blends emphasizing thermal resistance.

The degradation coefficients for m-Aramid content below 40% and above 60% are close to those for p-Aramid 100% and m-Aramid 100%, respectively. In the case of fabrics with high p-Aramid blends, when the p-Aramid blended yarn broke, the lower blended m-Aramid yarn could not withstand the load and broke. As a result, the degradation coefficient of p-Aramid became dominant. On the other hand, in the case of a fabric with a high m-Aramid blend ratio, even if the p-Aramid, which deteriorates rapidly, got damaged, the m-Aramid withstands it due to the low p-Aramid blend ratio. As a result, the degradation coefficient of m-Aramid would be dominant. The results imply that the synergistic effect of p-Aramid and m-Aramid blends on the degradation coefficient are slight and divided to the degradation coefficient of p-Aramid and m-Aramid. 

### 3.5. Case Study with the Predictive Equation for Retention Fraction of Tensile Strength

Consider Sample G’s retention fraction of tensile strength (m-Aramid 90%/p-Aramid 10%) an outer layer of commercially available firefighter clothing. The following equation gives a fitting equation for Sample G’s retention fraction of tensile strength.

(4) $$\;\frac{I}{I_{0}}=\exp\left(-0.48\sqrt{\frac{Q}{Q_{0}}}\right)\;\;\;\;\;\;\;\;\left(\;0\;\leq \;\frac{Q}{Q_{0}}\;\leq \;10\;\right)$$

$$\frac{I\;}{I_{0}}=\exp\left(-0.48\sqrt{\frac{Q}{Q_{0}}}\right)=\frac{450}{1490.7}$$

(5) $$\frac{Q}{Q_{0}}={\left(-\frac{\ln\frac{450}{1490.7}}{0.48}\right)}^{2}=6.2\;$$

The tensile strength (*I*) of the firefighter clothing for calculating the retention fraction of tensile strength was the tensile strength that the firefighter clothing should have (*I* = 450 N, performance level b1), as specified by ISO 11999-3 [5]. Since the fraction of UV exposure energy (*Q*/*Q*_0_) is equal to the number of years of UV exposure, the calculated number of years of UV exposure is 6.2 years by Equation (5) under the condition that the initial strength (*I_0_*) of specimen G is 1490.7 N and the tensile strength (*I*) is less than 450 N.

One of the four replacement conditions by NFPA 1851 [20] is when the firefighter clothing has reached the manufacturer’s suggested service life, typically 5 to 10 years. Therefore, the results of this case study are included in the service life indicated by NFPA 1851. Thus, for the range of p-Aramid and m-Aramid blended fabrics used in this study, the degradation coefficient for the blend ratio of p-Aramid and m-Aramid can be calculated as the ratio of UV exposure energy (*Q*/*Q*_0_) to any fraction of tensile strength (*I*/*I*_0_) or any UV exposure energy (*Q*/*Q*_0_) or the retention fraction of tensile strength (*I*/*I*_0_) for an arbitrary UV exposure energy ratio (*I*/*I*_0_). Therefore, the model equation can predict the aging of tensile strength of outer layers of firefighter clothing blended with p-Aramid and m-Aramid for the fabric masses used in this study.

## 4. Conclusions

This study focused on the UV degradation of the outer layers of firefighter clothing blended with p-Aramid and m-Aramid. It investigated the effects of blend ratio, fabric specification, and UV exposure energy on the tensile strength of the outer layers of firefighter clothing. UV exposure of nine fabrics of firefighter clothing was conducted using an accelerated degradation test apparatus, assuming that the clothing would be used for a maximum of 10 years. The UV exposure energy per year was determined based on interviews with firefighters. The results of tensile strength tests before and after UV exposure showed that the decrease in tensile strength due to UV degradation depended on the combination of p-Aramid and m-Aramid blend percentages. Tensile strength for all blend ratios decreased exponentially with UV exposure energy and was within the range of results for fabrics with p-Aramid 100% and m-Aramid 100%. The water repellent treatment did not affect tensile strength for fabrics with the same blend ratio, fabric mass, and fabric structure. The effect of fabric mass on tensile strength was significant for UV exposure energies from 0 MJ/m^2^ to 68 MJ/m^2^. Still, it decreased with increasing UV exposure energy for UV exposure energies above 102 MJ/m^2^.

For all fabrics used in this study, the retention fraction of tensile strength (*I*/*I*_0_) decreased exponentially as the fraction of UV energy ratio (*Q*/*Q*_0_) increased. Fitting the data of the retention fraction of tensile strength to the UV exposure energy ratio, the degradation coefficients *α_f_* for Sample A (p-Aramid 100%) and Sample H (m-Aramid 100%) were 0.73 and 0.51, respectively. The degradation coefficients *α_f_* for all fabrics used in this study showed within this range. Therefore, the experimental results could be classified into two groups based on m-Aramid blends of 40% or more and 60% or less. The number of years of service was calculated based on the desired strength, which generally agreed with the number of years of service indicated by NFPA 1851. The use of this model is expected to make it possible to easily predict the UV characteristics of fabrics of firefighter clothing that retain high mechanical strength through calculation and to design the tensile strength of firefighter clothing considering aging deterioration.

## Figures and Tables

**Table 1:** Specification of the test fabric.

Sample	Fiber and Blending Ratio (%)	Mass of Fabric (g/m^2^)	Number of Ripstop	Water Repellency
A	p-Aramid = 100	206	2	Yes
B	m-Aramid/p-Aramid = 20/80	235	3	No
C	m-Aramid/p-Aramid = 40/60	235	3	No
D	m-Aramid/p-Aramid = 60/40	235	3	No
E	m-Aramid/p-Aramid = 80/20	235	3	No
F	m-Aramid/p-Aramid = 90/10	252	2	No
G	m-Aramid/p-Aramid = 90/10	252	2	Yes
H	m-Aramid = 100	214	2	Yes
I	m-Aramid = 100	235	3	No

**Table 2:** UV exposure conditions.

Estimated Years (Year)	0	1	2	3	5	6	10
UV exposure energy (MJ/m^2^)	0	34	68	102	170	204	340
Exposure time (hour)	0	52.4	104.8	157.2	262.0	314.4	524.0

**Table 3:** Tensile strength and the retention fraction of tensile strength.

		UV Exposure Dosage (MJ/m^2^)						
	*Q* (MJ/m^2^)	0	34	68	102	170	204	340
	*Q*/*Q*_0_	0	1	2	3	5	6	10
A	*I* (N)	5208.9	2556.2	1870.4	1325.0	1019.0	901.0	619.2
	s (N)	28.6	234.8	182.5	46.5	52.1	59.0	45.8
	*I*/*I*_0_ (-)	1	0.49	0.36	0.25	0.20	0.17	0.12
B	*I* (N)	4596.3	2446.7	1670.5	1317.5	1029.0	898.3	573.0
	s (N)	123.7	76.5	9.1	29.5	5.7	20.9	35.9
	*I*/*I*_0_ (-)	1	0.53	0.36	0.29	0.22	0.20	0.12
C	*I* (N)	4223.2	2334.3	1559.0	1239.7	922.5	786.1	528.1
	s (N)	32.6	65.0	57.1	62.6	74.4	21.6	3.0
	*I*/I_0_ (-)	1	0.55	0.37	0.29	0.22	0.19	0.13
D	*I* (N)	2222.0	1446.1	1024.2	914.1	681.9	634.5	473.7
	s (N)	32.6	16.0	19.6	24.4	13.1	42.0	8.5
	*I*/*I*_0_ (-)	1	0.65	0.46	0.41	0.31	0.29	0.21
E	*I* (N)	1929.2	1334.0	1060.9	811.5	670.4	575.9	488.3
	s (N)	64.8	24.7	30.1	21.3	16.3	17.5	4.3
	*I*/*I*_0_ (-)	1	0.69	0.55	0.42	0.35	0.30	0.25
F	*I* (N)	1490.7	917.0	708.2	613.5	521.1	495.0	374.6
	s (N)	9.1	39.7	12.7	16.5	13.3	10.6	6.1
	*I*/*I*_0_ (-)	1	0.62	0.48	0.41	0.35	0.33	0.25
G	*I* (N)	1455.8	878.7	704.8	541.0	508.1	485.5	433.3
	s (N)	11.4	14.0	27.5	11.7	19.4	14.0	11.4
	*I*/*I*_0_ (-)	1	0.60	0.48	0.37	0.35	0.33	0.30
H	*I* (N)	1404.4	745.1	607.0	557.6	464.3	461.1	391.8
	s (N)	30.6	17.7	20.0	5.9	13.3	9.3	7.8
	*I*/*I*_0_ (-)	1	0.53	0.43	0.40	0.33	0.33	0.28
I	*I* (N)	1536.4	912.8	701.7	583.4	487.0	451.4	368.7
	s (N)	45.7	29.0	18.1	23.9	25.1	12.2	15.5
	*I*/*I*_0_ (-)	1	0.58	0.42	0.39	0.29	0.29	0.22

**Table 4:** Difference of tensile strength affected by water repellency (Samples F and G).

		UV Exposure Dosage (MJ/m^2^)						
	*Q* (MJ/m^2^)	0	34	68	102	170	204	340
	*Q*/*Q*_0_	0	1	2	3	5	6	10
F	*I* (N)	1490.7	917.0	708.2	613.5	521.1	495.0	374.6
	*I*/*I*_0_ (-)	1	0.62	0.48	0.41	0.35	0.33	0.25
G	*I* (N)	1455.8	878.7	704.8	541.0	508.1	485.5	433.3
	*I*/*I*_0_ (-)	1	0.60	0.48	0.37	0.35	0.33	0.30
$I_{F}-I_{G}$	(N)	−34.9	−38.3	−3.4	−72.5	−13.0	−9.5	58.7
$\frac{I_{F}-I_{G}}{I_{G}}$	(%)	−2.4	−4.4	−0.5	−13.4	−2.6	−2.0	13.5
${\left[I/I_{0}\right]}_{F}-{\left[I/I_{0}\right]}_{G}$	(-)	0	0.02	0	0.04	0	0	−0.05

**Table 5:** Difference of tensile strength affected by mass of fabric (Samples H and I).

		UV Exposure Dosage (MJ/m^2^)						
	Q (MJ/m^2^)	0	34	68	102	170	204	340
	*Q*/*Q*_0_	0	1	2	3	5	6	10
H	I (N)	1404.4	745.1	607.0	557.6	464.3	461.1	391.8
	*I*/*I*_0_ (-)	1	0.53	0.43	0.40	0.33	0.33	0.28
I	*I* (N)	1536.4	912.8	701.7	583.4	487.0	451.4	368.7
	*I*/*I*_0_ (-)	1	0.58	0.42	0.39	0.29	0.29	0.22
$I_{I}-I_{H}$	(N)	132.0	167.7	94.7	25.8	22.7	−9.7	−23.1
$\frac{I_{I}-I_{H}}{I_{H}}$	(%)	8.6	18.4	13.5	4.4	4.7	−2.1	−6.3
${\left[I/I_{0}\right]}_{I}-{\left[I/I_{0}\right]}_{H}$	[-]	0	0.05	−0.01	−0.01	−0.04	−0.04	−0.06
